# Multiple scale model for cell migration in monolayers: Elastic mismatch between cells enhances motility

**DOI:** 10.1038/srep11745

**Published:** 2015-07-02

**Authors:** Benoit Palmieri, Yony Bresler, Denis Wirtz, Martin Grant

**Affiliations:** 1 Department of Physics, McGill University, 3600 University, Montréal, Québec, Canada H3A 2T8; 2 Department of Chemical and Biomolecular Engineering and Johns Hopkins Physical Sciences-Oncology Center, The Johns Hopkins University, Baltimore, Maryland; 3 Johns Hopkins Physical Sciences - Oncology Center, The Johns Hopkins University, Baltimore, Maryland

## Abstract

We propose a multiscale model for monolayer of motile cells that comprise normal and cancer cells. In the model, the two types of cells have identical properties except for their elasticity; cancer cells are softer and normal cells are stiffer. The goal is to isolate the role of elasticity mismatch on the migration potential of cancer cells in the absence of other contributions that are present in real cells. The methodology is based on a phase-field description where each cell is modeled as a highly-deformable self-propelled droplet. We simulated two types of nearly confluent monolayers. One contains a single cancer cell in a layer of normal cells and the other contains normal cells only. The simulation results demonstrate that elasticity mismatch *alone* is sufficient to increase the motility of the cancer cell significantly. Further, the trajectory of the cancer cell is decorated by several speed “bursts” where the cancer cell quickly relaxes from a largely deformed shape and consequently increases its translational motion. The increased motility and the amplitude and frequency of the bursts are in qualitative agreement with recent experiments.

In many physiological processes, cells migrate by moving through narrow channels defined by the surrounding environment. One example is cancer metastasis, where a cancer cell squeezes through the endothelium to reach the blood stream and eventually forms a secondary tumor elsewhere in the body^1^,^2^,^3^,^4^. Over recent years, the study of cancer from a physical sciences point of view has drawn much attention^3^,^5^,^6^,^7^,^8^,^9^,^10^: Physical principles are believed to offer an alternative perspective of the disease and may help to optimize treatments^11^ and detection^12^. The model we present in this paper emphasizes the role of the elastic properties of cancer cells and surrounding normal cells on the metastatic potential of the former. Our simulations show that elasticity mismatch *alone* can reproduce features of cancer cell migration observed in experiments.

More precisely, we propose a multiple scale model to study the motility of individual cells in a larger cells-on-substrate assembly that comprises normal and cancer cells. We will focus on the nearly confluent scenario which describes monolayers. Understanding the behavior of cell monolayers is an important biological question that goes beyond the physics of cancer since epithelial tissues, which support the structure of embryos and organs, often have a monolayer structure^13^. Examples of cells-on-substrate experiments that are not directly related to cancer include studies of collective behavior^14^,^15^, wound healing^9^,^16^,^17^ and colony growth^18^.

Our work is motivated by recent experiments performed by Lee *et al*.^9^ which showed that the Young modulus of metastatic human breast carcinoma cells (MDA-MB-231) is about *three times smaller* than the one of human breast epithelial cells (MCF10A). In the same study, the authors showed that the motility of a cancer cell embedded in a confluent monolayer of mostly normal cells was much larger than in the case where the layer is made of cancer cells only. This observation was partly attributed to the fact that short speed “bursts” decorate the trajectory of the cancer cell. These bursts typically occur when a cancer cell, highly deformed due to temporary crowding by the neighboring normal cells, rapidly relaxes to a less deformed shape as the cell escapes the crowded configuration. Hence, it was proposed that the elasticity mismatch between cancer cells and normal cells significantly contributes to the observed “bursty” migration behavior and the concomitantly larger motilities of the cancer cells.

In the experiments, the increased motility of the metastatic cancer cells is probably due to many factors where one is the cell mechanical properties. Additional differences between cancer and normal cells include inter cellular adhesions^9^ and protrusion activity^19^. Here, the model parameters will be chosen so that all cells in the monolayer have identical properties except for their elasticity: Cancer cell are softer, normal cells are stiffer. The main results of our simulation studies demonstrate that elasticity mismatch alone is sufficient to trigger bursty migration behavior and significantly increase the motility of the soft cell. Moreover, the simulated migratory behavior of cancer cells in a layer of mostly normal cells is in qualitative agreement with the experiments^9^.

The model that we use permits the description of very large cell shape deformations. We will show that this point is crucial to accurately describe bursty migration. The effect of deformability of cells and vesicles has recently been studied in other contexts. Many of these studies were based on a beads-and-springs model for the cell shape and focused on red blood cells in capillaries^20^,^21^, bacteria in biofilms^22^,^23^ and tissue growth^24^. Such models complement recent Potts model studies of cell sorting^25^ and vertex model dynamical studies^26^,^27^ of soft tissues.

The phase-field model that we propose is more general than these other approaches. First, it can be easily extended to include more complexity (i.e., cell internal degree of freedom). Second, the inactive part of the dynamics is self-consistently derived from non-equilibrium thermodynamics principles. In that sense, our approach more closely resembles that used in Refs 6,7, which focused on tumor growth, and that used in Refs 28, 29, 30, 31, 32, 33, which focused on single cell behavior. Our phase-field model approach is applied to an *assembly* of cells and it retains shape and motion details at the single cell level. Modelling the system behavior down to the single cell level is important to describe the cooperation between normal and cancer cells that leads to the bursty migration behavior and the increased motility of the latter.

The paper is organized as follows. The next section contains the Results. The first subsection gives a brief summary of our cell monolayer model. Simulations results are given in the next two subsections. The second one reports the cell arrangement predicted by our model for monolayers of inactive (non-migrating) cells. These determine the initial conditions for the simulations of motile cells presented in the third subsection. There, the migratory behavior of a tagged cell in monolayers of varying cell mechanical properties is analyzed. Following is the Discussion. It contains a summary of our findings and discusses avenues to be explored. Finally, the Methods section gives extra details on the statics and dynamics aspects of the model and it gives a brief overview of the numerical procedure.

## Results

**Figure 1:**
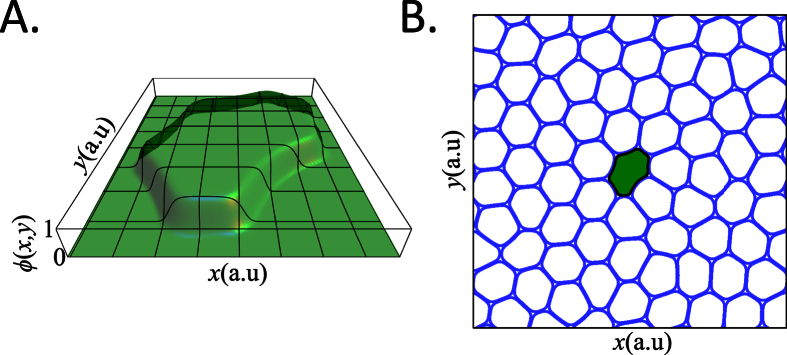
An example of a model monolayer comprising one soft cancer cell and normal cells. Each cell is described by a field, *ϕ*(*x*, *y*), that is defined to be 0 outside the cell and 1 inside. The field rapidly varies in the region of the cell boundary. **A**. The field of a single cancer cell. **B**. The monolayer is reconstructed by showing the boundary of all cells (Blue curves). A tagged cell, the cancer cell, is filled in Green with a Black boundary.

We model monolayers that comprise normal and cancer cells using a phase-field approach similar to the one recently employed by Najem *et al*.^30^, who studied chemotropism in neural cells, by Löber *et al*.^31^, who studied cell crawling on soft substrate, and by Larazo *et al*.^32^,^33^, who studied the shape of red blood cells under flow in small channels. Here, we treat the monolayer as a 2D system. This is a good level of description since the cells dynamics are constrained in a region close to the plane defined by the substrate. In our model, it is assumed that cells do not grow nor divide. The idea is that each cell is described by a “field” which rapidly varies in the region of the cell boundary. Hence, we denote by *ϕ*_*n*_(*x*,*y*; *t*) the field associated with cell *n* where *x*,*y* are the two spatial coordinates and *t* is the time. An example of a cell field is shown in Fig. 1A. The interior of each cell is defined by regions where *ϕ*_*n*_ = 1 while the exterior is defined by regions where *ϕ*_*n*_ = 0. At the cell boundary, *ϕ*_*n*_ interpolates rapidly between 0 and 1. From now on, all monolayer images that we will present only show the boundary of each cell except for a tagged cell that will be highlighted with a color as in Fig. 1B.

Details of the model are presented in the Methods section and in the Supplementary Information. However, a brief overview is given in the next subsection.

### Model outline

The most important advantages of our phase-field monolayer model are: 1) The cell boundary does not have to be tracked explicitly. 2) Extremely large deformations can be described. 3) The mechanical properties and the velocities of each cell can be modeled individually. The latter point is particularly important since one of our goals is to make connections with the experiments of Lee *et al*.^9^. Our approach differs from vertex models^26^,^27^ by allowing all types of cell deformations and any degree of cell coverage. The difference between our phase field model and an equivalent Cells Potts model^25^ is at the level of the dynamics since the former is the continuum limit of the latter.

The time dependent behavior of the monolayer is described by dynamical equations for the cell fields,


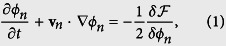


where 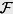 is the monolayer free energy, **v**_*n*_ is the translational velocity of cell number *n* and *δ* denotes a functional derivative. Note that this equation is written in terms of dimensionless units, which will be used throughout the paper. The relationships between dimensionless and real units are detailed in the Supplementary Information, briefly reviewed at the end of the Methods section. The right-hand side of Eq. (1) describes cell shape dynamics, which are determined by free energy changes. Details of the model free energy are given in the Methods section and in particular, by Eqs (7) and (10). The free energy contains several parameters and its minimum determines the prefered state of the system. Briefly, one parameter controls the elastic response of each cell to shape deformation (*γ*_*n*_ in Eq. (7)). Another controls the preferred radius of the cells (*R* in see Eq. (7)), which tend to be circular when they are not perturbed by other cells. Also, there is a parameter that controls the energy penalty for overlapping cells (*κ* in Eq. (10), which is chosen to be large). Note that the interactions between cells are strictly repulsive.

The velocity of each cell is the sum of two distinct contributions as described in Eq. (11): 1) The inactive part, **v**_*I*_, is due to the interaction force exerted by the other cells. Like the cell shape dynamics, the inactive part of the velocity is determined by free energy changes. 2) The active, or self-propulsion, part of the velocity, **v**_*A*_, is due to the cell engine. The relative strength of the two contributions to the velocity is determined by another parameter (*ξ* in Eq. (12)) which also controls the maximum cell shape deformation. The active part of the velocity is chosen so that the motion of isolated cells on the substrate is described by a persistent random walk^34^,^35^ where the characteristic cell speed and the reorientation statistics match the experimental observations^9^. Further, we assume that there is a large separation of time scales between the cell shape relaxation (fast) and the cell translation dynamics (slow). This approximation is physical since 1 *μm* deep indentations on cells typically relax within seconds^36^ and the motile cells we model translate by 1 *μm* within minutes^9^.

To highlight the role of cell elasticity mismatch, we considered 2 types of cell monolayers assemblies: a single cancer cell in a layer of normal cells (hereafter referred as the “soft-in-normal” case) and normal cells only (hereafter referred as the “all-normal” case). We performed simulations at two packing fractions, *ρ* = 0.85 and 0.9, describing nearly confluent monolayers, where,


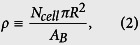


where *A*_*B*_ is the area of the simulation box. Overall, we will present a total of 4 simulations, each of which contains *N*_*cell*_ = 72 cells. All model parameters are listed in Table 1 and explained in details in the Methods section and in the Supplementary Information. Our aim is to isolate the effect of the mechanical properties of motile cells on their migratory behavior. Hence, *all parameters will be identical for both types of cells with the exception of the parameter which controls the cell stiffness* (we set *γ*_cancer_/*γ*_normal_ = 0.35, as observed experimentally). That includes the sequence of random numbers which determines the initial positions of the cells and the reorientation events. Hence, in the absence of elasticity mismatch and at the same packing fraction, the soft-in-normal and all-normal simulations would give identical results.

### Aging

In the first stage of all simulation, the cells are randomly placed in the simulation box at the appropriate packing fraction. All cells are initially circular with radius *R* given in Table 1. Any cell whose center is within a distance smaller than *R* to the center of a previously placed cell is randomly re-assigned to a new position. At a given packing fraction, the initial position of the cells for the soft-in-normal and all-normal cases are enforced to be the same. In the initial configuration, the monolayer is far from equilibrium since many cells overlap. Hence, we allow it to relax by numerically propagating Eq. (1) *without* the term that gives rise to self-propulsion, **v**_*n*,*A*_ in Eq. (11). During this “aging” period, the system rearranges to minimize the free energy and the cells act as mutually immiscible “dead” droplets.

**Figure 2:**
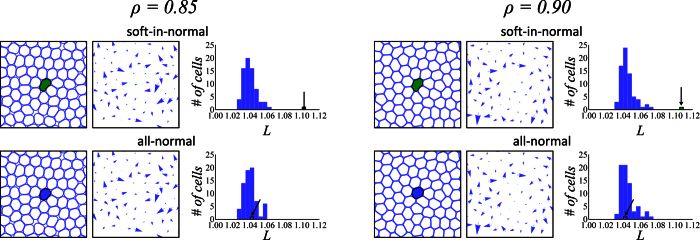
Summary of the aging simulations. The results of the four aging simulations are shown as groups of three panels. The left panel in a group shows the monolayer configuration after aging. The tagged cell is filled in Green (Blue) when it is a soft cancer (normal) cell. All other cells are normal and their boundaries are shown in Blue. The middle panel shows the displacement of each cell relative to its initial position. The right panel shows the distribution of the cell perimeter, *L*_*n*_ (see Eq. (3)), after aging. The arrow points toward the tagged cell. The left and right groups of three panels respectively correspond to the packing fractions *ρ* = 0.85 & 0.9 and the top and bottom groups respectively correspond to the soft-in-normal and all-normal cases.

Figure 2 shows the final configuration of all 4 monolayers after aging and the net displacement of each cells. A tagged cell is highlighted with a black boundary and a colored filling. This cell always starts in the middle of the simulation box. This is the cancer cell in the soft-in-normal simulations. The soft cancer cell is colored in Green and normal cells in Blue. The arrows that report the displacement of the cells during aging are colored in the same way. The length of the arrows is equal to *twice* the cell displacements magnitude. Movies of all 4 aging simulations are given in the Supplementary Information. Note that the cell displacements do not seem to correlate with the elasticity, but rather on the initial nearest-neighbor configuration of each cell. This is most easily seen in the Supplementary Movies. All supplementary movies are available at *http://dx.doi.org/10.6084/m9.figshare.1439474*. On the other hand, the deformation of the tagged soft cell in the soft-in-normal case is clearly larger than that of the other cells and that of the tagged normal cell in the all-normal case.

The distortion of each cell can be characterized by the length of their interface, or cell perimeter. With the cell field *ϕ*_*n*_ given by Eq. (S2), it is simple to show that the perimeter is proportional to the following measure,


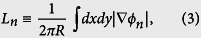


which is equal to 1 for perfectly circular cells. Because the cell area is nearly conserved, the cells can only reduce their interactions energy by deforming their boundaries. The perimeter increases with increasing cell deformation. Figure 2 also shows the distribution of *L*_*n*_ values at the end of the 4 aging simulations for all 72 cells. Note that the value of the tagged cell has been singled out and it is indicated by an arrow. Comparing the two rows that correspond to the soft-in-normal and the all-normal cases clearly shows that the soft cell undergoes deformations that are significantly larger than the deformation of the surrounding normal cells. At the end of the all-normal aging simulations, the system is probably close to a metastable state which differs from true equilibrium. In that latter case, the distribution of *L*_*n*_ values in the bottom row of Fig. 2 (all-normal simulations) would show a single peak since the minimum free energy state of the system has a hexagonal symmetry where all cells have the same perimeter. This equilibrium state may not be attainable, even with long simulation times.

### Motile cells

**Figure 3:**
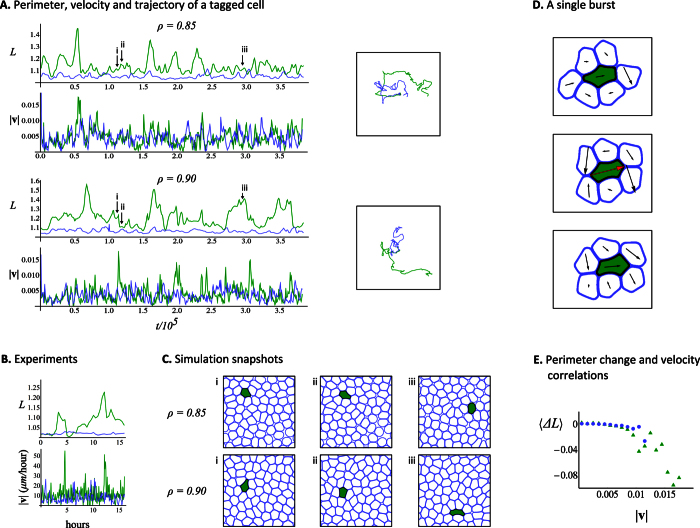
Summary of motile cells simulations. **(A)** The perimeter and instantaneous velocity, *L* and 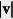, of the tagged cell as a function of dimensionless time. Green corresponds to the soft cancer cell (soft-in-normal simulations) and Blue corresponds to its normal counterpart (all-normal simulations). The top (bottom) two panels report the simulation results obtained with *ρ* = 0.85(0.90). The total simulation time corresponds to ≈40 hours when converted back in real time (see the Methods section). The two boxes show the trajectories of the tagged cells at the two packing fractions. **(B)** Experimental results reproduced with permission from Ref. 9 for a monolayer that comprises very few cancer cells and mostly normal cells. The value of *L* is calculated from the deformation of the cell nucleus and the total observation time is 16 hours. **(C)** Snapshots of the motile soft-in-normal monolayer simulations at *ρ* = 0.85 (top) and *ρ* = 0.90 (bottom) that correspond to the times indicated by the labels i, ii and iii in part A. **(D)** Three snapshots of the soft-in-normal simulation at *ρ* = 0.90 are shown at times just before, during, and right after the speed burst right after the label iii in part A. Only the soft cancer cell (filled in Green) and its neighbors (Blue boundaries) are shown. The length of the Black arrow on top of each cell is proportional to its instantaneous velocity. When collective effects between cells induce a speed burst to the soft cancer cell (i.e., when its instantaneous velocity is larger than the active part), the arrow is shown in Red. **(E)** Average change in cell perimeter, Δ*L*, between succesive time steps binned according to the cell instantaneous velocity, 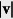. Note that part A and D have accompanying supplementary movies.

We now present the results obtained for motile cells simulations that correspond to the case where the velocity of each cell is given by Eq. (11) *including* the self-propulsion term, **v**_*n*,*A*_. The final configurations after aging shown in Fig. 2 are used as initial conditions for the motile cell simulations. Figure 3 summarizes the motile cell simulations and is the main result of our work. It clearly shows that cell elasticity mismatch plays a significant role on the cells dynamics.

Again, we will focus on the tagged cell. The color scheme is the same as before; the Green curves correspond to the soft cancer cell (soft-in-normal case) and the Blue curves correspond to the tagged normal cell (all-normal case). The path traced by the soft cancer cell embedded in the soft-in-normal simulations largely differs from the path traced by its normal cell counterpart in the all-normal simulations. This is shown in the square boxes in Fig. 3A. In particular, the space explored by the soft cancer cell exceeds that of its normal counterparts.

Second, the soft cancer cell undergoes very large deformations (see the time evolution of *L* in Fig. 3A) compared to the tagged normal cell at the same packing fraction. More precisely, in the soft-in-normal case, the perimeter of the soft cell reaches *L* = 1.5 which is 150% that of an undeformed cell. This contrasts the all-normal case where the same cell is normally stiff and its perimeter does not exceed *L* = 1.1.

Third, the instantaneous velocity of the soft cancer cell contains several spikes that correspond to short speed bursts. These are shown in the time evolution of 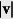 in Fig. 3A. A comparison with the evolution of *L* shows that these bursts occur simultaneously with a rapid decrease of the cell perimeter; as the cell rapidly relaxes to a lower energy, more circular, configuration. Bursts are clearly observed in the bottom part of Fig. 3A (and the corresponding Supplementary Movie) for the soft-in-normal case at the largest packing fraction. Note that, at the two packing fractions considered, the tagged cell does not display any speed bursts in the all-normal case. In this case, increasing the packing fraction simply reduces the mean instantaneous velocity of the normal cell.

These observations qualitatively reproduce many of the experimental features reported by Lee *et al*.^9^ for confluent monolayers of live cells. Their experimental data is reported in Fig. 3B, with permission. In the experiment, 2 bursts where observed in a 16 hours time window. Converted back to real units (see Sec. 0.3), the simulation results shown in Fig. 3A corresponds to a ≈40 hours time window during which 6–8 instantaneous velocity spikes (bursts) are observed. Of course, a statistically meaningful comparison between the simulation and experimental results cannot be done due to the small number if bursts observed experimentally and theoretically. However, Fig. 3A,B show that the simulations at *ρ* = 0.90 and the experiments are strikingly similar. One crucial point of our work is that the *only* difference between the cases considered, at fixed packing fraction, is in the cell elastic properties. Hence, our results support the hypothesis of Lee *et al*.^9^ that cell elasticity mismatch *alone* can enhance the motility of the softer cells.

Fig. 3C shows snapshots of the soft-in-normal simulation for *ρ* = 0.85 (top panels) and *ρ* = 0.90 (bottom panels). The snapshots are reported at times labeled by the marks i, ii and iii in Fig. 3A, which respectively corresponds to a time before the soft cancer cell undergoes a speed burst, after the speed burst and at a later time when the soft cell is largely deformed for the *ρ* = 0.90 case. Note that, at any time shown for the lowest packing fraction snapshots, the soft cell does is not largely deformed and many “empty spaces” are seen in the simulation box.

To further investigate the mechanism that leads to bursty migration, we used the soft-in-normal simulation at the largest packing fraction to generate supplementary movies that show the soft cancer cell, its nearest neighbors and their respective instantaneous velocities. Figure 3D reports three frames; before, during and after the speed burst that occurs right after the time marked by iii in Fig. 3A. The Black arrow in Fig. 3D shows the instantaneous velocity of the cells. It turns Red when the velocity magnitude of the soft cancer cell is larger than the magnitude of the active part alone, which is the speed the soft cell would have if it was isolated. Further, the figure shows that the increase in velocity is in part due to the fact that two neighboring cells effectively pinch the rear part of the cell, which is opposite to its velocity, and thereby increase its forward motion. In fact, the two rear-end cells undergo a T1 topological swap; a processes which has been observed in cell rearrangement in tissues^37^ and in the relaxation of topological defects in 2D^38^.

We next analyze in more details the behavior of the tagged cell for the simulations where the effect of the elasticity mismatch is more apparent (for *ρ* = 0.90). Simulations at that packing fraction were run for a much longer time (*t* = 2.0 × 10^6^ which corresponds to ≈200 hours in real time). These longer runs are used to perform a meaningful statistical analysis of the instantaneous velocity of the tagged cell. Figure 3E illustrates the correlation between cell perimeter change and instantaneous velocity. The long simulations are used to bin the perimeter change Δ*L* (between succesive times) according to the instantaneous velocity of the cell, 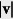. The figure reports 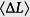, the average perimeter change in each velocity bin. Hence, negative Δ*L* corresponds to a cell that relaxed by decreasing its perimeter. The tagged normal cell (Blue circles) does not achieve the largest velocities of the tagged cancer cell (Green triangles). More importantly, the largest velocities of the cancer cell correlate with a large decrease in cell perimeter, particularly at 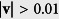, which is the magnitude of the self-propulsion speed.

**Figure 4:**
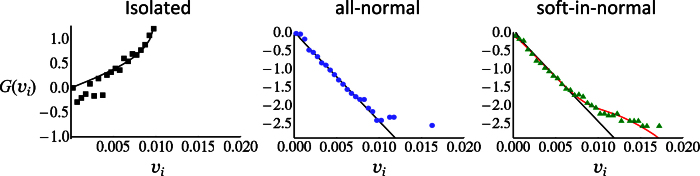
Velocity distribution of a tagged cell. The probability distribution of the *x* or *y* component of the instantaneous velocity of the tagged cell when the cell (soft or normal) is isolated on the substrate (left panel), in the all-normal case (middle panel) and in the soft-in-normal case (right panel). The distribution functions have been scaled such that a Gaussian distribution gives a straight line. For the Gaussian case, 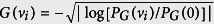. The markers show the simulation data. In the isolated case, the Black curve is the exact velocity distribution that arises from the active part of the velocity alone (see Eq. (4)). In the all-normal case, the velocity distribution of the tagged cell is well described by a Gaussian fit (Black line) with standard deviation *σ*_*G*_ = 0.0029. In the soft-in-normal case, the velocity distribution of the soft cancer cell is not well described by a Gaussian fit (Black line) with *σ*_*G*_ = 0.0028. It is better described by the distribution proposed in the main text, Eq. (5), which is shown here as the Red curve.

Further, the long runs were used to calculate probability distributions for the *x* and *y*-components of the instantaneous velocity of the tagged cell in the soft-in-normal and all-normal cases. The results are shown in Fig. 4. The first panel of Fig. 4 shows the probability distribution (scaled such that a Gaussian distribution is a straight line) of the active part of the cell velocity which is the velocity the cell would have if it was alone on the substrate. The curve is the exact result,


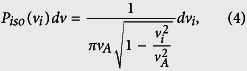


where *i* = *x* or *y* and the marks are the numerical results. Note that the distribution of the active part of the velocity is extremely non-Gaussian. However, the second panel in Fig. 4 shows that, for the all-normal case, the self-averaging induced by the interactions with the other cells transform the instantaneous velocity distribution into a Gaussian one, *P*_*G*_(*v*_*i*_) where the standard deviation of the distribution, *σ*_*G*_, is a fitting parameter.

On the other hand, the last panel in Fig. 4 shows that a Gaussian fit (Black line) cannot describe the instantaneous velocity distribution of the soft cancer cell in the soft-in-normal case (see the last panel in Fig. 4). Note that the simulation data has long tails (i.e., higher probability for large velocities) that are not accounted for by the Gaussian fit. We propose that the soft cancer cell is in one of two regimes. In the first and most probable one, it behaves like a normal cell and its velocity is described by a Gaussian. In the other, the cell undergoes bursty migration and the self-propulsion adds to the Gaussian contribution of the velocity. Mathematically, this is described by


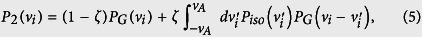


where *ζ* represents the fraction of time that the soft cancer cell is bursty. The right panel of Fig. 4 shows the fit of the data with this 2-regimes model (Red curve) where the standard deviation of *P*_*G*_ was obtained from the all-normal simulations and hence, *ζ* is the only fitting parameter which we found to be *ζ* = 0.038. This implies that the soft cell is in the bursty regime ≈3–4% of the time, in qualitative agreement with Fig. 3A. Note the point near v_*i*_ ≈ 0.015 in Fig. 3B is the only one that is significantly outside of the Gaussian distribution. In fact, this point is due to a single speed burst observed for the tagged normal cell at *ρ* = 0.9. In comparison, the soft tagged cell at the same packing fraction shows about 40 speed bursts.

Of course, other distributions with a longer than Gaussian tail can fit the soft-in-normal data in Fig. 4. In particular, the Student-t distribution^39^ with *β* = 7 degrees of freedom gives an equally good fit. The Student-t distribution arises when *β* + 1 samples are drawn from a Gaussian distribution of unknown variance. We find it intriguing that the number of degrees of freedom, *β* = 7, that fits our data is very close to the mean coordination number of each cell. We think that this may be due to the fact that the soft cell in the soft-in-normal case only “feels” its immediate nearest neighbors whereas in the all-normal case, all cells feel each other.

**Figure 5:**
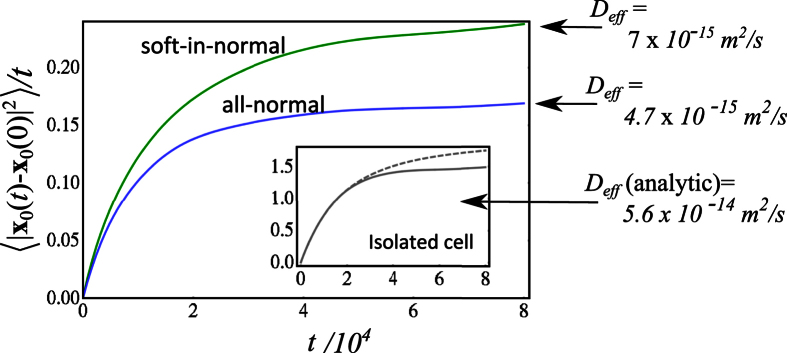
Motility of motile cells in monolayers. The mean-squared displacement of the tagged soft cancer in the soft-in-normal simulation (Green curve) and of the normal tagged cell in the all-normal simulation (Blue curve) are reported at the largest packing fraction (*ρ* = 0.90). The effective diffusion coefficients that characterize the migration potential of the tagged cell were calculated from the long-time limit of the mean-square displacement and converted back to real units. The inset shows the mean-squared displacement calculated from the simulation of a cell isolated on the substrate (full curve) and compares it with the analytical prediction (dashed curve) given by Eq. (14).

The motilities of the tagged cells can be extracted from their trajectories. More precisely, the mean-squared displacement (see Eq. (14)) of any tagged cell can be used to calculate an effective diffusion coefficient. Figure 5 shows the mean-squared displacement of the tagged cell for the long simulations at *ρ* = 0.90 which was computed using a time averaging procedure,


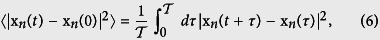


where 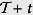 must be smaller than the total simulation time (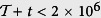). The curves in Fig. 5 are smooth, which is due to the fact that the average is performed over a long time interval. The effective diffusion coefficients that are reported in the figure are obtained from the long-time part of the curves in Fig. 5. The tagged soft cancer cell in the soft-in-normal simulation has the larger diffusion coefficient, which is about a factor of 1.5 larger than the one of the tagged normal cell in the all-normal simulation. Also note that the inset of Fig. 5 compares the analytical prediction of the mean squared-displacement given by Eq. (14) with the one calculated from the trajectory that the tagged cell would have if it was alone on the substrate for the same simulation time. The analytical result gives *D*_*eff*_ (isolated) = 5.6 × 10^−14^ *m*^2^/*s* which is about one order of magnitude larger than the effective diffusion coefficient for the tagged soft cancer cell or normal cell in a dense monolayer. The comparison with the numerical calculation shows that the latter gives an effective diffusion coefficient which is about 10% smaller than the analytical prediction. This discrepancy is due to the finite sampling of reorientation events. Note that it is particularly difficult to sample the tail of the exponential distribution that governs the reorientation statistics (see. Eq. (13)). This issue probably also affects the calculated diffusion coefficients for the tagged cells in monolayers reported in Fig. 5 which may in fact have slightly larger values if the simulations were run much longer. However, the important point here is the *relative* values of *D*_*eff*_ for the soft-in-normal and all-normal cases.

## Discussion

In this paper, we proposed a multiple scale model to describe cell dynamics in monolayers with any degree of confluence. Results obtained with the model focused on a nearly confluent monolayer comprising of cancer cells and normal cells. Our main goal was to assess the role of cell elasticity mismatch on the migration potential of the cells (i.e., the metastatic potential of the cancer cells), in light of the recent experimental studies of Lee *et al*.^9^ who observed that human breast carcinoma cells (MDA-MB-231) embedded in a monolayer of mostly normal human breast epithelial cells (MCF10A) displayed an increased motility in comparison to the case where they are alone on the substrate. This larger migration potential was partly attributed to the fact that the cancer cells, whose Young modulus is about 3 times smaller compared to the normal cells, can deform their shape easily and squeeze between neighboring normal cells leading to bursty migration. The simulation results obtained with the model treated cancer and normal cells identically, except for their elastic constant. This allowed us to quantify the role of elasticity mismatch *alone*. The most important results that we obtained are: 1) Our minimum energy model shows that the motility (quantified by an effective diffusion coefficient) of a soft cancer cell in a monolayer of normal cells can be 50% larger (see Fig. 5) as the one of a normal cell in the monolayer. 2) The trajectory of the soft cell in a layer of normal cells is decorated by speed bursts, where the velocity significantly deviates from its average value, in qualitative agreement with the experimental observations. 3) The velocity distribution of the soft cell in a layer of normal cells shows longer tails that are inconsistent with a Gaussian distribution. Of course, in our simulations, elasticity mismatch is solely responsible for these effects.

Several speed bursts are shown in the bottom two panels of Fig. 3A (Green curve). We have further observed that most bursts are induced by a T1 topological swap where two normal cells, initially separated by the cancer cell, move toward each other and connect by pinching the rear-end of the cancer cell. This process gives a net push to the cancer cell (see Fig. 3D and the corresponding Supplementary Movie) which rapidly relaxes to a more circular shape. We observed 6–8 bursts in the soft-in-normal simulation at the highest packing fraction (*ρ* = 0.90) over a time scale that correspond to ≈40 hours in real time. Lee *et al*.^9^ reported 2 bursts over an observation period of 16 hours (see Fig. 3B). Of course, the small number of bursts prevents us to compare the frequency/amplitude of our bursts with the experimental ones in a statistically meaningful way. Nevertheless, the qualitative agreement between the experiments and our simulations at *ρ* = 0.90 is striking. Moreover, our simulations allows us to quantify the correlation between the instantaneous velocity of the tagged cell and the instantaneous change in cell perimeter (see Fig. 3E). A calculation shown in Sec. S6 of the Supplementary Information further shows that most bursts can be described as short events were the tagged cell moves with the velocity it would have if it was alone on the substrate *plus* an extra contribution that arises from the cell displacement due to the rapid cell shape change (i.e., when a highly deformed tagged cell rapidly relaxes to a more circular shape).

To some degree, our results are sensitive on the choices of the model parameters. In particular, for the lowest packing fraction considered, the soft cancer cell does not show clean speed bursts. This observation seems to suggest that confluence is required for bursty migration. There is much more empty space between cells in the *ρ* = 0.85 simulations compared to the *ρ* = 0.90 (compare the top and bottom rows of Fig. 3C and the Supplementary Movies). Further note that *ρ* does not necessarily correspond to the cell packing fraction in the real system. The difference arises from the fact that in the model, the cell is an elastomer defined by a single elastic constant. On the other hand, the elastic restoring force that prevents real cells from migrating through narrow pathways primarily comes from the nucleus since the cytoplasm is much softer^40^. Future studies will be performed to quantify the role of packing fraction more precisely. If our results are sensitive to the choice of *model parameters*, they should not be sensitive to the choice of the model. In fact, we believe that our results could have been obtained using a Cell Potts description^25^.

One important difference between our work and the one of Lee *et al*.^9^ is the following. Recall that our goal was to isolate the role of cell elasticity. Hence, we set the active, self-propulsion, part of the velocity of all cells to be the same. However, in the experiment, that does not seem to be the case. In fact, the cancer cell appears to move much less by itself than the normal cells^9^. Hence, when it is embedded in a layer of normal cells, its motility may primarily be increased because it gets pushed around by the other, more motile cells. In our simulations, the strength of the self-propulsion of the cancer and normal cells is identical. Hence, when any type of cells are brought together in a dense monolayer, to first order, their motility decreases since the cells act as obstacles to each other. In the future, we want to study the effect of active velocity mismatch to understand how a soft cell that is embedded in a layer of normal cells (with a different active velocity magnitude) could cross over from the case where it is more (less) motile in the monolayer in comparison to the case where it is alone on the substrate.

Another difference between our simulation studies and the experiments of Lee *et al*. is that cell-cell adhesion and more importantly, how it varies with cell type, was neglected. In fact, the experiment showed that the magnitude of the bursts is largest when normal cells adhere with each other, but not with the soft cancer cell^9^. Cell-cell adhesion can be included in our phase field monolayer model, but this goes beyond the objective of the current work. We are planning to include it in future extensions of the model. Note that in real monolayers, the integrity of a cluster of cells is maintained through cell adhesion. In our model, the cells remain clustered due to the boundary conditions. Hence, our model includes adhesion in its simplest form.

One important advantage of the phase-field description for monolayers of motile cells that we propose is that it scales favorably with the number of fields one may want to add to each cells to include details that we left over for simplicity. A natural example are fields that describe the density of actin and myosin in the cytoplasm or focal adhesions on the cell surface which can generate protrusive forces, contractile forces and strain in the substrate^41^,^42^. Such cell internal degrees of freedom have been included in recent 2D models of a single migrating cell^28^,^29^,^31^. Including new fields that describe such cell internal processes does not increase the computational effort significantly, as long as they do not require using a finer mesh. Generalizing our model to 3D may also help to understand why motile cells appear to migrate using drastically different mechanisms on 2D substrates compared to the case when they are embedded inside a 3D collagen matrix^35^,^44^,^45^. Phase-field models can be extended from 2D to 3D, but the concomitant increase in numerical cost usually requires the use of more sophisticated numerical methods.

## Methods

### Model free energy

We treat each cell as a 2D soft body for which the equilibrium shape minimizes the following free-energy,


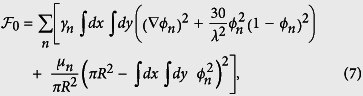


where it is understood that, by *ϕ*_*n*_, we mean *ϕ*_*n*_(*x*,*y*; *t*). The form of Eq. (7) guarantees that the preferred values for the cell fields are *ϕ*_*n*_ = 0 and 1. The length over which *ϕ*_*n*_ varies from 0 to 1, *λ*, corresponds to the width of the boundary of any cell. In the model, non-interacting cells tend to be circular with a radius *R*. Energetic costs associated to changes in cell area are determined by *μ*_*n*_. We next show that *γ*_*n*_ is the parameter that controls the elasticity of the cells.

We calculated the energy cost predicted by the model and that results from a sinusoidal deformation of the cell boundary (see Sec. S1 of the Supplementary Information). For a deformation wavelength equal to 2*πR*/*k*, where *k* is a wavenumber, and an amplitude equal to *ε*, the energetic cost is


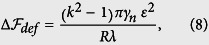


where periodicity imposes that *k* is an integer larger than 1 and where we assume that *ε*/*R* ≪ 1. For identical cell sizes, interface widths and deformations, 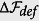 depends on cell type only through *γ*_*n*_. Hence, *γ*_*n*_ is the parameter that controls the cell stiffness. Note that the energy cost due to shape deformation can scale with the wavenumber, *k*, with a different power law if the curvature energy is taken into account^46^ (in which case it scales like *k*^4^) or if the cell interior is treated as an elastic medium^47^ (in which case it scales like 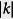). Here, these contributions are neglected since our focus is not on the details of the underlying restoring force.

We now comment on the last term in Eq. (7) which constrains the area of the cell. In the simulations, *μ*_*n*_ will be chosen to be large enough so that the cells will prefer to deform when they are brought together rather than shrink. It is not crucial that the cell area be exactly conserved in our description of cell monolayers. In reality, it is the cell volume which should be conserved for cells that neither grow nor divide. In our 2D model, the cell area can be interpreted as the area of a 3D cell “projected” onto the substrate. This projected area can deviate from its preferred value as long as the cell thickness above the substrate is simultaneously adjusted such that the overall cell volume remains constant^25^,^26^.

The free energy given by Eq. (7) describes each cells individually. The total free energy of the monolayer is,


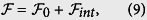


where


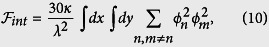


describes the interaction between cells that prevents them from overlapping. Both *ϕ*_*n*_ and *ϕ*_*m*_ are non-zero in a region where cells *n* and *m* overlap and the resulting energy cost is controlled by *κ* (like *μ*_*n*_, *κ* will be chosen sufficiently large so that cells will deform when they are brought together rather than overlap). In terms of *ϕ*, Eq. (10) is the lowest order term that gives the desired repulsion between cells. More details on the model parameters and their meaning are given in the Supplementary Information.

### Dynamics

The motion of each cell within the monolayer is described by Eq. (1). The time-dependent cell velocity, **v**_*n*_, is chosen to be spatially uniform for simplicity. Our model fall into the category of “model A” in the classification scheme of Hohenberg and Halperin^48^ for dynamical critical phenomena, but with an extra convective term. The right-hand-side of Eq. (1) determines how rapidly a deformed cell returns to its circular, equilibrium shape. Such a relaxation time scale can be determined from cell viscosity measurements (for a recent example, see Ref. 36) or can be estimated by equating the shape relaxation rate with the viscous dissipation of the water inside the cell (see Ref. 49).

The velocity of each cell is divided in two parts,


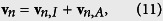


where **v**_*n*,*I*_ and **v**_*n*,*A*_ are the inactive and active (self-propulsion) parts of the velocity of cell *n*, respectively. The inactive part is due to forces exerted on cell *n* by the other cells while the self-propulsion part is due to internal processes that require energy consumption. The constitutive equations for **v**_*n*,*I*_ can be determined from thermodynamic principles. With **v**_*n*,*A*_ = 0, the free energy of the system, which is an assembly of dead deformable droplets, should be a strictly decreasing function of time. In Sec. S2 of the Supplementary Information, we use this condition to show that,


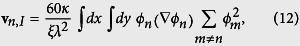


where *ξ* is interpreted as the friction between the cells, the substrate and the surrounding water. Together with Eq. (1), the last equation guarantees that the monolayer will tend toward thermodynamic equilibrium in the absence of active forces. At this point the cells do not flow, **v**_*n*,*I*_ = 0, and their boundaries do not move, 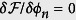.

Of course, live cells never reach thermodynamic equilibrium. This is taken into account by 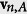 which is chosen such that the instantaneous velocity of isolated cells has a constant magnitude; 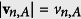. On the other hand, the time dependence of the cell motion is due to the orientation of 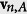 which we describe as a random process where the time interval between reorientation events, 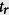, follows an exponential distribution given by,


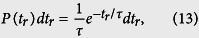


where *τ* is the average time between two reorientations. Hence, in our model, an isolated cell moves in a straight line with constant speed between two reorientation events. Eq. (13) can also be used to calculate the mean-squared displacement of cell *n* when it is isolated on the substrate,


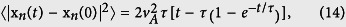


where x_*n*_(*t*) is the center of cell *n* and 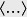 implies an average over an ensemble of isolated cell trajectories or equivalently, a longer time average over a single isolated cell trajectory as given by Eq. (6). The long time behavior 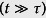 of the mean-squared displacement is linear in time and hence, isolated cells can be characterized by an “effective” diffusion coefficient in two dimensions, 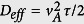. Rather than depending on temperature, the effective diffusion coefficient depends on internal processes that require energy consumption and that determine *v*_*A*_ and *τ*.

### C. Model Parameters and Numerical Considerations

All model parameters are chosen to be identical for both types of cells with the exception of *γ*_*n*_. The simulation will be performed by propagating Eq. (1) numerically on a uniform mesh. Table 1 summarizes the model parameters used in the simulations. As stated in the Results section, the parameters are obtained from the experiments or by invoking physical approximations. Additional details are given in Sec. S4.

**Figure 6:**
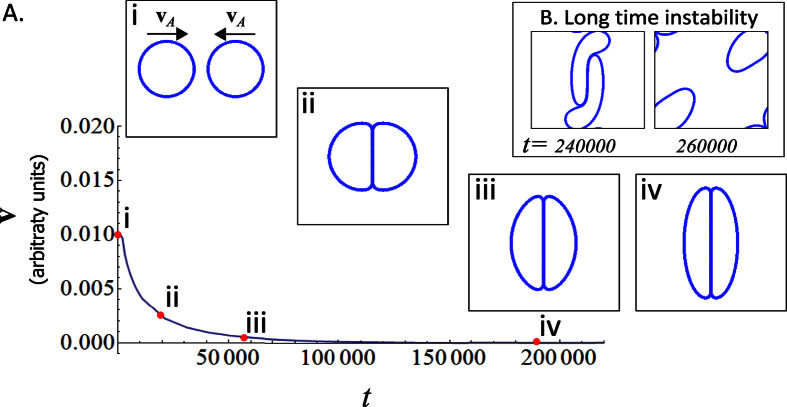
Simulation results for two normal cells that move toward each other with opposite velocities. The parameter *ξ* that controls the relative strength of the active force, **v**_*A*_ and the interaction force, **v**_*I*_, is chosen to be *ξ* = 1.5 × 10^3^. **(A)** The *x*-component of the dimensionless *total* velocity of the left cell is reported as a function of time. Snapshots of the cells configuration are given at four times. **(B)** At long times, the *y*-component of the inactive part of the velocity deviates away from zero due to small numerical errors. This causes the cells to deviate from their original direction and pass by one another, deforming their shapes in the process.

The determination of *ξ* requires further comments. Consider two cells that move toward each other in an “head on collision” manner so that they have a constant and opposite active force parallel to their separation vector, see Fig. 6. As the two cells start interacting/touching, a) they deform and b) they decelerate. Hence, the cells will reach a conformation where the force term due to interactions with the other cells exactly cancels the active force so that **v**_*I*_ + **v**_*A*_ = 0. For increasing *ξ*, cells need to be increasingly deformed for the interaction forces to decrease the velocities. Figure 6 reports the total velocity of one cell for the head-on collision just described with *ξ* *=* 1.5 × 10^3^ (as listed in Table 1). The long-time maximum deformation of the cells is in qualitative agreement with the largest cell deformation observed in monolayers. Note that the two cells end up in a metastable configuration from which they can escape at long time due to numerical error build-up.

Fig. 6 has an accompanying supplementary movie.

Simulations of the monolayer model are performed in a square simulation box of area *A*_*B*_ = *N*_*mesh*_ × *N*_*mesh*_ with *N*_*mesh*_ the number of mesh points along one axis of the box. Eq. (1) is integrated on a mesh, periodic boundary conditions are used and additional details of the numerical procedure are given in the Supplementary Information.

Importantly, our simulation results can be converted back to real units using the following simple arguments. The cells we are modeling have a radius of the order of 10 *μm* and our cells have a radius of 49 mesh points. Hence, the distance between mesh points in our simulations is ≈0.2 *μm*. In the experiment, the average instantaneous velocity of normal cells in a confluent monolayer of mostly normal cells is ≈10 *μm*/*hour* (see Fig. 3B). Alone on the substrate, it should be larger since the motion of any given cell is not blocked by the others. Hence, we assume that *v*_*A*_ = 20 *μm*/*hour* which means that *t* = 1 in our simulations is equivalent to 0.36 *s* in real time.

## Additional Information

**How to cite this article**: Palmieri, B. *et al*. Multiple scale model for cell migration in monolayers: Elastic mismatch between cells enhances motility. *Sci. Rep*. **5**, 11745; doi: 10.1038/srep11745 (2015).

## Supplementary Material

Supplementary Information

Supplementary Movie S1

Supplementary Movie S2

Supplementary Movie S3

Supplementary Movie S4

Supplementary Movie S5

Supplementary Movie S6

Supplementary Movie S7

Supplementary Movie S8

Supplementary Movie S9

Supplementary Movie S10

## Figures and Tables

**Table 1:** Numerical values used for the dimensionless model parameters. Normal and Cancer cells only differ in the parameter that controls their stiffness, *γ*_*n*_.

Dimensionless parameter	*γ*_*n*_=		*λ*=	*R*=	*κ*=	*μ*=	*τ*=	*v*_*A=*_	*ξ*=
numerical value	0.35	1	7	49	10	1	10^4^	10^−2^	1.5 × 10^3^
Cell type	cancer	normal	all						
