# Supplementary material for: Multiple scale model for cell migration in monolayers: Elastic mismatch between cells enhances motility
Source: Sci Rep. 2015 Jul 2;5:11745. doi: 10.1038/srep11745 (PMC5155609; doi:10.1038/srep11745)
Supplement: Supplementary Information [file srep11745-s1.pdf]

Supplementary Information for : "Multiple scale model for cell migration in monolayers: Elastic mismatch between cells enhances motility"

Benoit Palmieri,[1] Yony Bresler,[1] Denis Wirtz,[2,3] and Martin Grant[1]

[1] *Department of Physics, McGill University, 3600 University, Montréal, Québec, Canada H3A 2T8*

[2] *Department of Chemical and Biomolecular Engineering and Johns Hopkins Physical Sciences-Oncology Center, The Johns Hopkins University, Baltimore, Maryland*

[3] *Johns Hopkins Physical Sciences - Oncology Center, The Johns Hopkins University, Baltimore, Maryland*

S1. MONOLAYER FREE ENERGY

We start by writing the free energy of a single cell in the monolayer as,

$$\mathcal{F}_n = \int dx \int dy \ \left(\gamma_n(\nabla\phi_n)^2 + a_n\phi_n^2(1-\phi_n)^2\right) + \mu_n\left(\mathcal{A}_n - \int dx \int dy \ \phi_n^2\right)^2 , \tag{S1}$$

where the physical meaning of all parameters and variables is explained in the main text except that here, they have real units (in contrast to the dimensionless ones used in the main text). Further, a_n is a parameter that enforces that the preferred states for the cell fields are 0 and 1.

Consider perfectly circular cells modeled with the following profile,

$$\phi_n(r,\theta) = \begin{cases} \alpha_n & \text{for} \quad r < R_n - \lambda_n/2, \\ \frac{\alpha_n}{\lambda_n}\left(R_n + \frac{\lambda_n}{2} - r\right) & \text{for} \quad R_n - \lambda_n/2 < r < R + \lambda_n/2 \\ 0 & \text{for} \quad r > R_n + \lambda_n/2 \end{cases} , \tag{S2}$$

where R_n and λ_n are, respectively, the radius and thickness of cell n. $\phi_n = \alpha_n$ inside the cell and, in the interface region, ϕ_n decays linearly from α_n to 0 along the radial direction with respect to the cell center.

In the model, when a cell is not perturbed by the others, it has a circular shape with a radius $R_n^{(0)}$ (i.e., $\mathcal{A}_n = \pi {R_n^{(0)}}^2$ in Eq. (S1)). We will start by setting $\alpha_n = 1$ since we expect from the form of Eq. (S1) that this value for α_n minimizes the free energy. The cell profile given by Eq. (S2) is plugged in Eq. (S1) and the resulting single cell free energy is

minimized with respect to λ_n to show that the most favorable interface thickness of each cell is,

$$\lambda_n = \left(\frac{30\gamma_n}{a_n}\right)^{1/2}, \tag{S3}$$

which is valid for $\lambda_n \ll R_n^{(0)}$, i.e., the thickness of the cell boundary is much smaller that the cell radius. In Eq. (7), a_n was written in terms of γ_n and the interface width, λ_n.

Next, we allow for sinusoidal modulations of the cell boundary by letting $R_n \to R_n + \epsilon_n \cos k_n\theta$ in Eq (S2).This modified profile for ϕ_n is then inserted in Eq. (S3) and an analytical expression for the free energy is obtained:

$$\begin{aligned}\mathcal{F} &= \frac{\pi\gamma\alpha^2}{\lambda}\left\{2R + \frac{\epsilon^2 k^2}{R}\right\} + \frac{\pi a\alpha^2}{60}\left\{(1-\alpha)\left[30(1-\alpha)\epsilon^2 + (5-7\alpha^2)\lambda^2\right] - 4(5-15\alpha+9\alpha^2)\lambda R + 60(1-\alpha)^2R^2\right\} \\ &+ \frac{\nu}{\pi {R^{(0)}}^2}\left\{{R^{(0)}}^2 - \alpha^2\left(\frac{\epsilon^2}{2} + \frac{\lambda^2}{12} - \frac{\lambda R}{3} + R^2\right)\right\}^2, \end{aligned} \tag{S4}$$

where $\nu = \mu\pi{R^{(0)}}^2$. Note that in this last equation, the n subscript was omitted on all variables to keep the notation simpler. We want to know: 1) what are the values of R, α, λ and ϵ that minimize Eq. (S4), i.e., the equilibrium values for an isolated dead droplet, and 2) what is the free energy cost for small deviations about these equilibrium values. To do this, we write $R = R_{eq} + \delta R$ with similar expressions for α, λ and ϵ.

The equilibrium values of R, α, λ and ϵ are obtained by minimizing Eq. (S4). This is done perturbatively by assuming that R and $R^{(0)}$ are much larger than all other length scales. The result is,

$$\begin{aligned} R_{eq} &= R^{(0)} + \frac{2\lambda^{(0)}}{15} - \frac{\gamma}{2\lambda^{(0)}\nu} + O(\lambda^{(0)}{R^{(0)}}^{-1}), \\ \lambda_{eq} &= \lambda^{(0)} - \frac{7{\lambda^{(0)}}^2}{60R^{(0)}} + O({\lambda^{(0)}}^2{R^{(0)}}^{-2}) \\ \alpha_{eq} &= 1 + \frac{\lambda^{(0)}}{30R^{(0)}} + O({\lambda^{(0)}}^2{R^{(0)}}^{-2}) \\ \epsilon_{eq} &= 0, \end{aligned} \tag{S5}$$

where $\lambda^{(0)}$ is given by Eq. (S3). Of course, the last of these equations $\epsilon_{eq} = 0$ simply states that the cells prefer to be circular rather than deformed, as desired. The energy of a deformed cell (characterized by δR, $\delta\lambda$ $\delta\alpha$ and ϵ) is,

$$\begin{aligned}\mathcal{F}_{min} + \delta\mathcal{F} &= \frac{4\pi\gamma R^{(0)}}{\lambda^{(0)}} + \frac{\pi\gamma(k^2-1)}{\lambda^{(0)}R^{(0)}}\epsilon^2 + 4\pi\nu\delta R^2 + \frac{2\pi\gamma R^{(0)}}{{\lambda^{(0)}}^3}\delta\lambda^2 + \pi{R^{(0)}}^2\left(\frac{30\gamma}{{\lambda^{(0)}}^2} + 4\nu\right)\delta\alpha^2 \\ &- \frac{4\pi\nu}{3}\delta\lambda\delta R - 2\pi R^{(0)}\left(\frac{3\gamma}{{\lambda^{(0)}}^2} + \frac{2\nu}{3}\right)\delta\alpha\delta\lambda + 8\pi\nu R^{(0)}\delta\alpha\delta R. \end{aligned} \tag{S6}$$

The first term of this equation is the energy of an undeformed cell; $\mathcal{F}_{min} = 4\pi\gamma R^{(0)}/\lambda^{(0)}$. The second term describes the energy cost associated with small undulations of the cell boundary. This result is given in the main text, in dimensionless units, by Eq. (8). Note that the mode with the longest wavelength ($k = 1$) does not cost energy to that order, but one can show that this mode is stabilized by higher order terms in ϵ. The other terms represent the energy cost for compressing the cell (varying δR, $\delta\alpha$) or changing its interface thickness (varying $\delta\lambda$). Note that these other deformations cost much more energy (as indicated by the higher powers of $R^{(0)}$ in their prefactor) than the deformation that arises due to undulation of the boundary (as long as the cell deformation wavenumber, k, is not too large).

S2. MONOLAYER DYNAMICS

The motion of each cell within the monolayer is described by the following set of equations,

$$\frac{\partial \phi_n}{\partial t} + \mathbf{v}_n \cdot \nabla\phi_n = -\Gamma_n \frac{\delta \mathcal{F}}{\delta \phi_n}, \tag{S7}$$

where $\mathcal{F} = \mathcal{F}_{int} + \sum_n \mathcal{F}_n$, t is the time and δ denotes a functional derivative. Further, the interaction part of the free energy is given by,

$$\mathcal{F}_{int} = \int dx \int dy \sum_{n,m\neq n} \kappa\phi_n^2\phi_m^2. \tag{S8}$$

As explained in the main text, the time dependent and spatially uniform velocity of each cell can be divided in two parts,

$$\mathbf{v}_n = \mathbf{v}_{n,I} + \mathbf{v}_{n,A}, \tag{S9}$$

where $\mathbf{v}_{n,I}$ ($\mathbf{v}_{n,A}$) describes the inactive (active) part of the velocity. The derivation of the inactive part uses arguments similar to those of Wise et al. (see Appendix A of Ref. [1]) who focused on tumor growth. We start by writing Eq. (S7) as,

$$\frac{\partial \phi_n(x,y;t)}{\partial t} + \mathbf{v}_n(t) \cdot \nabla\phi_n = j(x,y;t), \tag{S10}$$

where $\mathbf{v}_n(t)$ and $j(x, y; t)$ are thermodynamic fluxes (see Ref. [2]). The goal is to identify constitutive relationships so that the system free energy strictly decreases as a function of time, $d\mathcal{F}/dt < 0$ for all t. The time derivative of the free energy can be written as,

$$\begin{aligned} \frac{d\mathcal{F}}{dt} &= \sum_n \int dx \int dy \, \frac{\delta F}{\delta\phi_n(x,y;t)} \frac{\partial\phi_n(x,y;t)}{\partial t}, \\ &= \sum_n \left[\int dx \int dy \, j(x,y;t) \frac{\delta F}{\delta\phi_n(x,y;t)} - \mathbf{v}_n(t) \cdot \int dx \int dy \, \frac{\delta F}{\delta\phi_n(x,y;t)} \nabla\phi_n(x,y;t) \right], \end{aligned} \tag{S11}$$

where the second line was obtained using Eq. (S10). From this last equation, it is simple to show that the constitutive equations,

$$\begin{aligned} j(x,y;t) &= -\Gamma_n \frac{\delta F}{\delta\phi_n(x,y;t)}, \\ \mathbf{v}_n(t) &= \xi_n^{-1} \int dx \int dy \, \frac{\delta F}{\delta\phi_n(x,y;t)} \nabla\phi_n(x,y;t), \\ &= \xi_n^{-1} \int dx \int dy \, 4\phi_n \left(\nabla\phi_n\right) \sum_{m\neq n} \kappa\phi_m^2, \end{aligned} \tag{S12}$$

ensure that $d\mathcal{F}/dt < 0$ for positive Γ_n and ξ_n. The last equality was obtained using Eqs. (S1) and (S8) for $\mathcal{F}$ (and made use of the fact that integrals of full derivatives vanish). This expression for $j(x, y; t)$, plugged into Eq. (S10) reduces to Eq. (S7) and the expression for $\mathbf{v}_n(t)$ also reduces to the one given in the main text, Eq. (12), when written in dimensionless form. More generally, the right-hand-side of the last equation describes the thermodynamic forces that induce the associated thermodynamic fluxes on the left-hand-side. In principle, cross-terms ($j(x, y; t)$ can be induced by the thermodynamic force associated to $\mathbf{v}_n(t)$) are thermodynamically allowed as long as they satisfy the Onsager reciprocal relations [2, 3]. Here, such cross-terms are neglected for simplicity.

Alternatively, we can treat the cells as particles so that their velocities obey Newton-like equations,

$$m_n \frac{\partial \mathbf{v}_n}{\partial t} = -\xi_n \mathbf{v}_n + \mathbf{f}_n^\dagger + \mathbf{F}_n, \tag{S13}$$

where ξ_n is the friction between the cell and the substrate, $\mathbf{f}_n^\dagger$ is a random force that arises due to internal processes that require energy consumption (i.e., the cell engine) and $\mathbf{F}_n$ is the force exerted on cell n by its neighbors. We will show that ξ_n right above is identical to the one introduced in Eq. (S12). Note that in Eq. (S13), $\mathbf{v}_n$ is the cell velocity given by Eq. (S9). For most biological applications, friction dominates inertia (see section 6.2 in Ref. [4]) so

that the acceleration term on the left-hand side of the last equation can be set to zero. Hence, it is simple to show $\mathbf{v}_{n,I} = \mathbf{F}_n/\xi_n$ and $\mathbf{v}_{n,A} = \mathbf{f}_n^\dagger/\xi_n$.

The force felt by any given cell, $\mathbf{F}_n$, as it moves in the presence of the other cells, can be determined by treating the free-energy, $\mathcal{F}$, as a potential,

$$\mathbf{F}_n \cdot \hat{e} = \lim_{\varepsilon \to 0} \frac{\mathcal{F}(\phi_n(\mathbf{x})) - \mathcal{F}(\phi_n(\mathbf{x} - \hat{e}\varepsilon))}{\varepsilon}, \tag{S14}$$

where $\hat{e} = \hat{x}$ or $\hat{y}$ is the positive unit vector along x or y, $\mathcal{F}(\phi_n(\mathbf{x}))$ is the free-energy of the system when all cells (including cell n) are at their actual position and $\mathcal{F}(\phi_n(\mathbf{x} - \hat{e}\varepsilon))$ is the energy of the system if cell n is translated by an amount ε along $\hat{e}$ with the other cells fixed. Simple manipulations of the last equation combined with the definition of $\mathbf{v}_{n,I}$ reproduces the solution shown above in Eq. (S12). This suggests that ξ_n in Eq. (S12) is a parameter describing the effective friction with the substrate. In the absence of self-propulsion (i.e. when $\mathbf{f}_n^\dagger = 0$), the cells behave as "dead" deformable vesicles which flow as a whole in a time-dependent manner with their velocities given by Eq. (S12).

S3. CONVERSION TO DIMENSIONLESS UNITS

In dimensionless units, the dynamics of the cell fields are governed by the following equations,

$$\begin{aligned}\frac{\partial \phi_n}{\partial \tilde{t}} &+ \tilde{\mathbf{v}}_n \cdot \tilde{\nabla}\phi_n = \tilde{\gamma}_n \tilde{\nabla}^2 \phi_n \\ &- \frac{30}{\tilde{\lambda}^2}\left[\tilde{\gamma}_n \phi_n (1-\phi_n)(1-2\phi_n) + 2\tilde{\kappa}\sum_{m\neq n}\phi_n\phi_m^2\right] \\ &- \frac{2\tilde{\mu}}{\pi\tilde{R}^2}\phi_n\left[\int d\tilde{x}\int d\tilde{y}\phi_n^2 - \pi\tilde{R}^2\right].\end{aligned} \tag{S15}$$

and

$$\tilde{\mathbf{v}}_n = \tilde{\mathbf{v}}_{n,A} + \frac{60\tilde{\kappa}}{\tilde{\lambda}^2\tilde{\xi}}\int d\tilde{x}\int d\tilde{y}\ \phi_n\left(\tilde{\nabla}\phi_n\right)\sum_{m\neq n}\phi_m^2. \tag{S16}$$

The tilde on top of any variable/parameter means that it is dimensionless and $\tilde{\nabla}$ implies a discrete derivative taken on the mesh. Note that in the main text, the dimensionless variables were written without the tilde, for simplicity. The real variables can be recovered in terms of Δ, the distance between neighboring mesh points, the reference cell

Model parameters	$\lambda =$	$R =$	$\gamma_n =$	$\kappa =$	$\mu =$	$t =$	$\mathbf{v_n} =$	$\xi =$
Expression	$\Delta\tilde{\lambda}$	$\Delta\tilde{R}$	$\gamma_0\tilde{\gamma}_n$	$30\gamma_0\tilde{\kappa}/\tilde{\lambda}^2\Delta^2$	$\gamma_0\tilde{\mu}/\pi\tilde{R}^2\Delta^4$	$\tilde{t}\Delta^2/2\Gamma\gamma_0$	$2\Gamma\gamma_0\tilde{\mathbf{v}}_n/\Delta$	$\tilde{\xi}/\Gamma$

TABLE I: The model parameters are expressed in terms of the dimensionless variables that appear in the dynamical equations, Eqs. (S15), and the distance between neighboring mesh points, Δ, the reference cell stiffness parameter, γ_0, and the mobility, Γ.

stiffness parameter γ_0 (the elastic constant of any *normal* cell), the mobility Γ and the relations given in Table I. Note that the dimensionless free energy is $\mathcal{F}/\gamma_0$.

S4. DETERMINING MODEL PARAMETERS

All model parameters are chosen to be identical for both types of cells with the exception of $\tilde{\gamma}_n$, which controls the cell stiffness. Hence, we now omit the subscript n for all parameters but the elasticity. The first two parameters, $\tilde{\lambda}$ and $\tilde{R}$, are chosen for numerical reasons. The smallest length scale in the system is the interface width of the model cells, $\tilde{\lambda}$. Variations on that length scale are described by 7 mesh points. The interface thickness does not play a role in the cell shape dynamics as long as it is much smaller than the cell radius [5]. Hence, we set $\tilde{R} = 7\tilde{\lambda}$.

Our choice of dimensionless units fixes the value of the elasticity parameter for the normal cells to $\tilde{\gamma}_n = 1$. The Atomic Force Microscopy Nano-indentations performed on live cells in Ref. [6] showed that metastatic cancer cells are about 3 times softer than normal cell. Hence we set $\tilde{\gamma}_n = 0.35$ for the cancer cells. The values for the parameters $\tilde{\kappa} = 10$ and $\tilde{\mu} = 1$ are chosen to enforce that configurations where cells overlap or shrink, respectively, are energetically costly compared to those where cells deform their boundary. The results of Sec. S1 and the definitions is listed in Table I shows that the energy cost for cell deformation (of wavelength k and amplitude $\tilde{\epsilon}$) and for cell compression/expansion (characterized by a change in radius $\delta\tilde{R}$) both depend linearly on $\tilde{\gamma}_n$. However, their ratio is given by $(\tilde{\epsilon}k/\delta\tilde{R})^2/4\tilde{\mu}\tilde{\lambda}\tilde{R}$. With the set of parameters we used (see the main text), that ratio can be written as $(\tilde{\epsilon}k/\delta\tilde{R})^2/1372$. Hence, for similar modulation and compression/expansion amplitudes (i.e for $\tilde{\epsilon} \approx \delta\tilde{R}$), it is much more easy to deform a cell (rather than changing its area) unless the modulation wavenumber is comparable to or larger than $k = 37$.

The experiments also showed that, on average, isolated cells move a distance equal to their size before changing directions. Hence, we choose $\tilde{\tau}\tilde{v}_A \approx 2\tilde{R}$. To determine $\tilde{\tau}$ separately from $\tilde{v}_A$, we assume that the characteristic time scale for cell shape rearrangements is small compared to the characteristic time scale for cell motion. This approximation is physical since $1\mu m$ deep indentations on cells typically relax within seconds [7] and the motile cells we model translate by $1\mu m$ within minutes [6]. This separation of scales is also required in our model that treats cells

as droplets. Without allowing sufficient time for shape relaxation, the cells could break up into smaller ones as they push against one another. The choice $\tilde{\tau} = 10^4$ and $\tilde{v}_A = 10^{-2}$ satisfies this separation of time scale requirement.

A simple calculation shows that cells rearrange their shapes on a time scale given by $\tilde{t}_{shape} \approx \tilde{R}^2/\tilde{\gamma}_n k^2$. Hence, the time it takes for a cell modulation with wavenumber k to decay divided by the time it takes for the same cell to move a distance equal to that modulation wavelength is approximately given by $\tilde{t}_{ratio} = \tilde{t}_{shape}/(\tilde{R}/k\tilde{v}_A) = \tilde{v}_A\tilde{\gamma}_n^{-1}(\tilde{R}/k)$. We chose $\tilde{\tau} = 10^4$ and $\tilde{v}_A = 10^{-2}$ (so that $\tilde{\tau}\tilde{v}_A \approx 2\tilde{R}$) which gives $\tilde{t}_{ratio} \approx 1/2\gamma_n k$. This is smaller than one for all cells and all wavenumber except $k = 1$. In our simulations, all cell deformation modes are "rapid" compared to cell translation except the longest wavelength mode, which evolves on a comparable timescale. Finally, as the cells move around in the presence of the other cells, the width of their boundaries will vary. On the other hand, time scale over which the boundaries recover their preferred thickness is exponentially fast and hence, is always much faster than the other time scales discussed above.

S5. DETAILS ON THE NUMERICAL SIMULATIONS

The simulations of the monolayer model were performed in parallel on a computer with 36 processors. Gradients on the mesh are taken using a simple two point formula while, for Laplacians, we use the isotropic formula due to McLellan [8]. Periodic boundary conditions at the edges of the simulation box are employed. Note that using periodic boundary conditions guarantees that the cells remain clustered. Without them, an initial cluster of cells will spread and effectively "dilute" the monolayer by increasing the average distance between cells. To speed the computation, fields that correspond to each of the cells are propagated in a smaller rectangular region of the simulation box. Initially, the size of this region is chosen to be $5R \times 5R$. Outside of that box, the field of the cell is assumed to be zero. The size, position and aspect ratio of each of these smaller boxes are updated as the cell moves toward its box boundary. At the start of the aging simulations, Eq. (S7) is propagated with a small time step $\delta t = 10^{-3}$ which is adaptively increased to $\delta t = 10^{-2}$ at later times. This latter time step is then used in the motile cells simulations.

S6. MAGNITUDE OF THE SPEED BURSTS

We hypothesize that most bursts can be described as short events were the tagged cell moves with the velocity it would have if it was alone on the substrate *plus* an extra contribution that arises from the cell displacement due to the rapid cell shape change. This can be tested using the following simple arguments. Consider an elliptical cell,

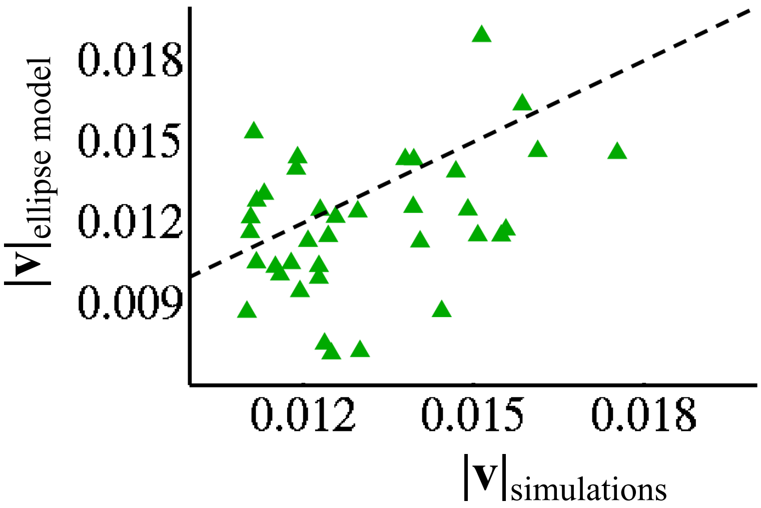

FIG. S1: The magnitude of speed bursts predicted by the simple model treating cells as ellipses (described in the main text) is compared with the simulated bursts. Velocities predicted by change in cell perimeter $\tilde{L}$ are compared to those observed in our simulations. The slope of the dashed line is equal to 1. If the model was perfect, all points would fall on the line. Note that only speed bursts with $|\tilde{v}| > 0.011$ are shown.

initially with major axis $\tilde{a}_I$ (subscript I stands for "initial") and an area $\tilde{A} = \pi\tilde{R}^2$. Using a first order approximation for the perimeter of an ellipse, its initial value can be written as $\tilde{L}_I = \frac{\sqrt{\tilde{a}_I^4+\tilde{R}^4}}{\sqrt{2}\tilde{R}\tilde{a}_I}$. The cell is allowed to relax toward a more circular shape, by keeping one side of the cell fixed (i.e., an obstacle which could be another cell). The final, more circular ellipse has a major axis $\tilde{a}_F < \tilde{a}_I$. This relaxation results in a net translation of the center of mass of the cell. Given the time interval between the initial and final configuration, $\Delta\tilde{t}$, this model predicts a velocity $v = \frac{(\tilde{a}_F - \tilde{a}_I)/2}{\Delta\tilde{t}} + \tilde{v}_A$. Fig. S1 compares the velocity prediction of the model with the simulation results, for every point where $|v| > 0.11$. The comparison is noisy, but nevertheless shows that the magnitude of the bursts predicted by the simple model is in qualitative agreement with the simulation results.

[1] S. Wise, J. Lowengrub, H. Frieboes, and V. Cristini, J. Theor. Biol. **253**, 524 (2008).

[2] S. de Groot and P. Mazur, *Non-equilibrium Thermodynamics* (Dover, 1984).

[3] L. Onsager, Phys. Rev. **37**, 405 (1931).

[4] V. Cristini and J. Lowengrub, *Multiscale Modeling of Cancer: An Integrated Experimental and Mathematical Modeling Approach* (Cambridge University Press, New York, 2010).

[5] K. Elder, M. Grant, N. Provatas, and J. Kosterlitz, Phys. Rev. E **64**, 021604 (2001).

[6] M. Lee, P. Wu, J. Staunton, R. Ros, G. Longmore, and D. Wirtz, Biophys. J. **102**, 2731 (2012).

[7] L. M. Rebêlo, J. de Sousa, J. M. Filho, J. Schäpe, H. Doschkeb, and M. Radmacher, Soft Matter **10**, 2141 (2014).

[8] J. McClellan, Proc. 7th Annual Princeton Conf. Information Sciences and Systems p. 247 (1973).
